# Identification and characterization of nonpolio enterovirus associated with nonpolio-acute flaccid paralysis in polio endemic state of Uttar Pradesh, Northern India

**DOI:** 10.1371/journal.pone.0208902

**Published:** 2019-01-30

**Authors:** Harjeet Singh Maan, Tapan N. Dhole, Rashmi Chowdhary

**Affiliations:** 1 Department of Microbiology, Sanjay Gandhi Post Graduate Institute of Medical Sciences, Lucknow, India; 2 Department of Biochemistry, All Indian Institute of Medical Sciences, Bhopal, India; University of Malaya, MALAYSIA

## Abstract

Despite polio eradication, nonpolio enterovirus (NPEV) detection amid polio surveillance, which is considered to have implications in paralysis, requires attention. The attributes of NPEV infections in nonpolio-AFP (NPAFP) cases from Uttar Pradesh (UP), India, remain undetermined and are thus investigated. A total of 1839 stool samples collected from patients with acute flaccid paralysis (AFP) from UP, India, between January 2010 and October 2011 were analyzed as per the WHO algorithm. A total of 359 NPAFP cases yielded NPEVs, which were subjected to microneutralization assay, partial VP1 gene-based molecular serotyping and phylogenetic analysis. Demographic and clinical-epidemiological features were also ascertained. Echoviruses (29%) and Coxsackievirus (CV)-B (17%) were the most common viruses identified by the microneutralization assay. The molecular genotyping characterized the NPEVs into 34 different serotypes, corresponding to Enterovirus (EV)-A (1.6%), EV-B (94%) and EV-C (5.3%) species. The rarely described EV serotypes, such as EV-C95, CV-A20, EV-C105, EV-B75, EV-B101, and EV-B107, were also identified. NPEV-associated AFP was more prevalent in younger male children, peaked in the monsoon months and was predominantly found in the central part of the state. The NPEV strains isolated in the study exhibited genetic diversity from those isolated in other countries. These form part of a different cluster or subcluster existing in cocirculation, limited to India only. This study augments the understanding of epidemiological features and demonstrates the extensive diversity exhibited by the NPEV strains in NPAFP cases from the polio-endemic region. It also underscores the need or effective long-term strategies to monitor NPEV circulation and its associated health risks in the post-polio eradication era.

## Introduction

Since 1988, global efforts led by the World Health Organization (WHO) have interrupted the transmission of indigenous wild poliovirus (WPV) and reduced the global incidence of poliomyelitis [1, 2]. With the success of the eradication program, wild polio virus (WPV), the most common cause of acute flaccid paralysis (AFP), has been eliminated from India. However, AFP continues to present as a major cause of neuromotor impairment in children in erstwhile polio-endemic areas [2, 3] in India, largely in adjacent states of Bihar and UP [4], where the annual prevalence of nonpolio AFP (NP-AFP) has consistently been higher than the global rates [2].

Nonpolio enteroviruses (NPEVs; family Picornaviridae) could potentially be the causative agents of many NP-AFP cases, as they are frequently detected and isolated during laboratory surveillance for poliomyelitis [5, 6]. NPEVs are associated with neurological illnesses and have been identified in AFP children [7–9]. However, only few reports of the isolation and epidemiology of AFP-associated NPEVs are available from India [10–13].

Accordingly, the goals of the present study were to identify and characterize epidemiological and genetic features of nonpolio enterovirus infections associated with AFP children in the erstwhile polio-endemic state of UP, the region that itself once represented a burden of more than 80% of global WPV AFP cases [14].

## Material and methods

### Ethics statement

This study constitutes a component of the Global Polio Eradication Initiative. This study did not involve human cases but did involve the use of cell culture isolates of viruses recovered from stool specimens collected from AFP patients through routine poliomyelitis surveillance activities. All ethical and technical features were approved by the WHO and the health authorities. The protocol and oral consent were part of routine surveillance activities recommended by the Steering Committee of the WHO and are consistent with all pertinent national regulations that protect human subjects. The methods complied with the principles of the Declaration of Helsinki. For collection of samples from healthy children, all the necessary approvals in accordance with the approved guideline of the Internal Review were obtained from the Ethics Review Committee of the Sanjay Gandhi Post Graduate Institute of Medical Sciences, Lucknow, India. Written informed consents were obtained from the parents or the legal guardian of the children.

### Study subjects, clinical samples and virus isolation

A total of 1839 AFP patients consisting of 1046 males and 793 females were included in the study, and their stool samples were collected from UP, India, during January 2010–October 2011. As part of the eradication program, two stool samples were collected ≥24 hours apart within 14 days of the onset of paralysis from each child <15 years of age who clinically manifested AFP symptoms as defined in the AFP surveillance program. Stool samples were stored at -70°C until processed. These were processed according to the WHO protocol [15]. The RD (human rhabdomyosarcoma) cell line and the L20B cell line (mouse fibroblast cells expressing the poliovirus receptor) were used for virus isolation, and their cytopathic effects (CPEs) were observed. The positive isolates with enterovirus (EV)-like CPEs were tested by pan-PV and pan-EV reverse transcription-polymerase chain reaction (RT-PCR) [15)]. All specimens from AFP patients that were positive for the polio virus (PV) and a mixture of PV and NPEVs according to the WHO algorithm were excluded from the analysis presented here. All AFP patients who had ≥1 stool specimen confirmed for NPEV had at least 1 specimen analyzed for NPEV typing and characterization. The clinical data and AFP-line lists of AFP patients were obtained from the National Polio Surveillance Project (NPSP) office, Lucknow. A total of 100 stool samples were also collected from healthy children without diarrhoea or any other symptom of illness. This control group shared a similar geographical area and socioeconomic status with those who manifested AFP.

### Serotyping, genotyping and molecular characterization

Identification of RD-positive isolates was performed for serotyping by a microneutralization technique (MNT) according to the WHO protocol [15]. The identity of the virus was confirmed using the specific antiserum, which prevented the appearance of CPEs. The isolates with no neutralization pattern were classified as untypable enteroviruses (UT-EVs).

The viral RNA was extracted from a stool suspension of all the UT-EVs and the negative isolates by using a Viral RNA Mini Kit (QIAamp, QIAGEN, Hilden, Germany). The molecular genotyping was performed by RT-PCR and nucleotide sequencing of a portion of the VP1 region as described by [16, 17]. In addition, some of the UT-EV isolates identified by VP1 sequencing with electropherograms showing superimposed peaks and thus suspected to contain mixed serotypes were resolved by inserting the PCR product into the pTZ57R/T cloning vector (2.8 Kb) using an Ins TA clone PCR Cloning Kit, according to the manufacturer's instructions.

**Fig 1:**
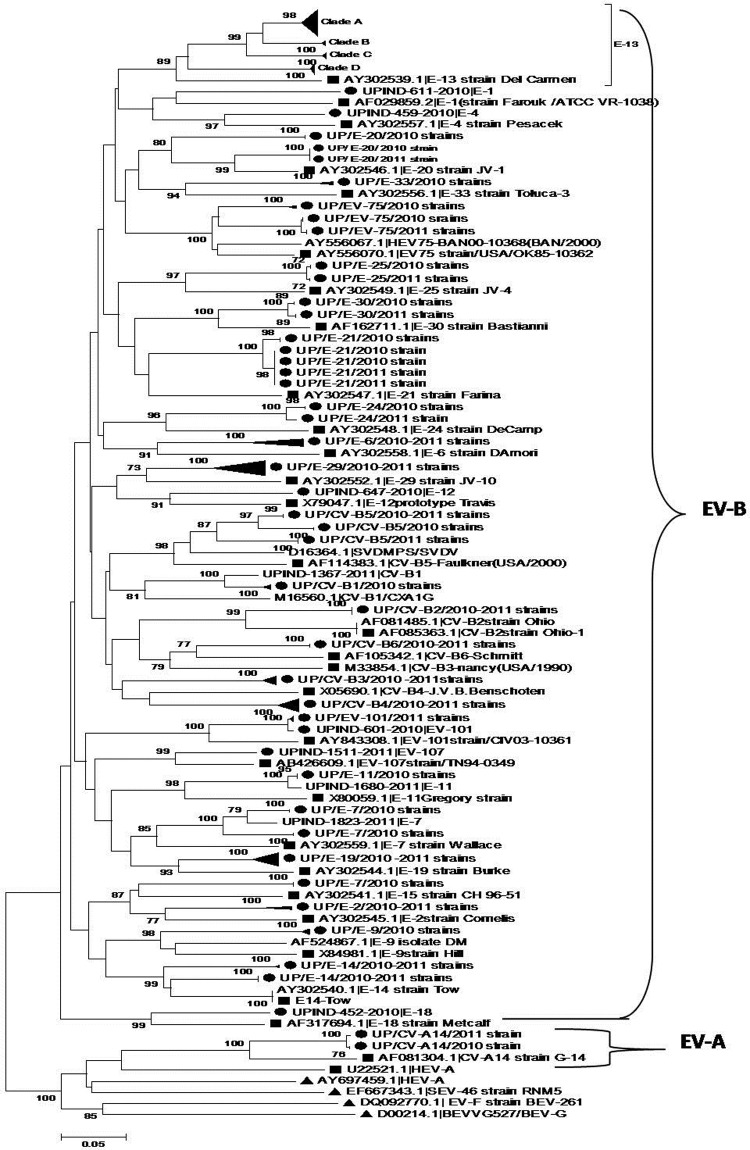
Phylogenetic analysis of partial VP1 region of EV serotypes of EV-B and EV-A species. (●), Black-closed circles represent NPEV seotypes from this study. (■), Black closed square box, represent all EV’s prototypes indicated by their accession numbers preceding serotype identification The tree was constructed by using the neighbor-joining method implemented in MEGA version 5.05. software, by using the Tamura-Nei’s nucleotide substitution model. The tree was evaluated with 1000 bootstrap pseudoreplicates. Bootstrap value >50% are indicated on the tree. Scale bar indicates genetic distance (measured in changes per nucleotide). The group, including the UP E-13 was condensed in to their respective clades, accordingly for clarity.

The partial VP1 nucleotide sequences of NPEV strains obtained from sequencing were compared with the EV reference strain sequences available in the GenBank database using a BLAST server (http://www.ncbi.nlm.nih.gov/blast) for serotype identification. MEGA 5.05 software (http://www.megasoftware.net) was used for phylogenetic analysis. To understand the genetic relatedness of isolates in India that belonged to an EV type to those from other countries, phylogenetic analyses of VP1 sequences of EV isolates from Uttar Pradesh that belonged to the prevalent E-13, CV-B4, E-19 and the numbered enterovirus EV-B75 types were performed to understand the representation of the VP1 sequences of the isolates of the corresponding serotypes into different clusters and subclusters within the types available in GenBank (Fig 1). A phylogenetic tree was generated by models of nucleotide substitution as mentioned in the legends of the related figures. The nucleotide sequences generated in this study have been deposited in the GenBank under accession numbers HM639724-HM639727, HQ023237-HQ023241, JN160782-JN160802, JN381481-JN381489 and KJ632519-KJ632655.

### Statistical analysis

The chi-square (χ^2^) test, independent samples t-test, regression analysis and Z-Test calculation for two population proportions were applied for statistical calculation where appropriate in the study by using SPSS for Windows version 16 (SPSS Inc, Chicago, USA).

## Results

### Serotyping and molecular typing

From the 1839 total AFP cases, 359 fecal specimens identified as putative EVs on the basis of CPEs in RD cells tested positive by pan-EV RT-PCR, and 5 EV RNA genomes were detected in stool suspensions of negative isolates. MNT enabled the serotypic identification of 163/359 (45%) RD-positive EV isolates into 16 different EV serotypes, mostly belonging to EV-B species (Table 1). Echoviruses (E) and Coxsackievirus B (CV-B) were the most common isolated serotypes. CV-B and E were isolated in 17% and 29% of the typed isolates, respectively. The remaining 196 (55%) yielded no neutralization pattern and were reported as UT-EVs.

**Table 1:** NPEV isolates identified by seroneutralization and molecular typing by VP1 PCR and sequencing.

EV isolates	EV antisera pool	VP1PCR and sequencing	Total
**Enterovirus-A**	-	3	**3**
CV-A14	-	3	3
**Enterovirus-C**	-	9	**9**
CV-A20	-	2	2
EV-C95	-	3	3
CV-A24	-	3	3
EV-C105	-	1	1
**Enterovirus-B**	163	176	**339**
CV-A9	1	-	1
CV-B	61	40^#^	101
E-1		1	1
E-2	1	2	3
E-3	1	-	1
E-4	3	2	5
E-6	14	5	19
E-7	4	8	12
E-9	6	4	10
E-11	14	5	19
E-12	10	1	11
E-13	19	37	56
E-14	5	6	11
E-15	-	3	3
E-18	-	1	1
E-19	-	9	9
E-20	3	6	9
E-21	-	7	7
E-24	-	3	3
E-25	8	7	15
E-29	9	10	19
E-30	4	3	7
E-33	-	2	2
EV-B75	-	8	8
EV-B101	-	5	5
EV-B107	-	1	1
Total	**163**^†^	**188**^‡^	**351**

Numbers in bold face represent the total number of NPEV isolate detected in EV-A, EV-C and EV-B species

^#^CV-B typed by VP1 PCR and sequencing as CV-B1 (n = 4), CV-B2 (n = 6), CV-B3 (n = 8), CV-B4 (n = 10), CV-B5 (n = 9) and CV-B6 (n = 3); n, represent the number of isolates.

^†^EV isolates typed by microneutralization

^‡^ UT-EV isolates characterized by molecular typing excludes isolates with mixed infections.

All 201 isolates, including 196 UT-EV and 5 EV RNA of negative isolates, were characterized by VP1 genotyping. A total of 188 EVs were genotyped by VP1-PCR and sequencing. The three isolates with suspected mixed infections were resolved by VP1-PCR followed by cloning and sequencing. Five serotypes, belonging to EV-B and EV-C species, were detected in mixed infections: CV-B5 and CV-B3, E- 11 and E-13, and CV-A21. All the NPEV serotypes identified in this study belong to the EV species-B (EV-B), EV-A and EV-C, with no EV-D species detected. The predominance of EV-B species (94%) and low levels of EV-A (1.6%) and EV-C (5.2%) species consisted of 34 serotypes (Table 1). Among the EV serotypes genotyped by VP1 sequencing, echoviruses were the predominant serotypes identified, followed by CV-B, CV-A belonging to EV-C and EV-A (EV-C: 9 and EV-A: 3), numbered EVs (EV-B75, EV-B101, EV-C95, EV-C105 and EV-B107) and the general rare serotypes such as CV-A14 and CV-A20 (Table 1).

Among the 34 serotypes, E-13 represents more than 19% (37/188) of the genotypically characterized isolates than other serotypes, followed by CV-B4 (10%) and E-29 (10%). The strains belonging to the 11 serotypes CV-B2, E-14, E-20, E-11, E-21, E-25, CV-B3, E-7, EV-B75, CV-B5, and E-19 each accounted for 3.3% to 4.5% of the characterized strains, and those belonging to the 7 serotypes CV-A14, CV-A20, CV-A24, CV-B6, E-15, E-24, E-9, CV-B1, E-6, E-30, and EV-B101 serotypes each represented 1.7% to 2.9%. The frequency of detection of each of the other serotypes, such as E-1, E-4, E-12, E-18, EV-C105, EV-B107, EV-C95, E-2, and E-33, ranged from 0.5% to 1.1%. The distribution of all the NPEV serotypes identified in this study reveals that E-13, E-6, E-7, E-11, E-25, E-29 and CV-B were found more frequently during the year 2010. However, E-6, E-7, E-11, E-12, E-13, E-14, E-19, E-20, E-21, E-25, E-29, E-30, EV-B75 and EV-B101 were identified during both 2010 and 2011, while E-1, E-4, E-15, E-18, and E-33 were identified and were more common in circulation during 2010 but not in 2011.

#### Identification of different NPEV serotypes in healthy children

Eighteen of 100 (18%) EVs were confirmed as NPEVs by pan-EV RT-PCR, and 10/18 (55%) NPEVs were typed by the neutralization method as CV-B (n = 4), E-11, E-12, E-13, E-25 (1 each) and E-6 (n = 2), whereas 8/18 (45%) remained untyped, and 7 were typed as CV-A20, E-13, E-19, CV-B3, CV-B4 and EV-B80 (1 each). No significant conclusion was inferred for the NPEVs associated with AFP patients when compared with the healthy children in terms of demographic characteristics such as gender, geographical distribution and seasonal variation, as the p value was >0.05 for comparisons among or between these variables. This was due to the low frequency of values for each of the variables in the control group of healthy children.

### Clinical and epidemiological features of AFP cases associated with NPEVs infection

Of 364 AFP patients, 207 (57%) were found to have fever at the onset of paralysis. However, no significant difference was observed in the number of AFP patients infected with CV-A or CV-B and Echo or numbered EVs (62/110, 56% *vs* 138/241, 57%: p>0.05) (Table 2). The occurrence of asymmetric paralysis was found significantly more in AFP patients infected with CV-A or CV-B than in those infected with Echovirus (E) or numbered EVs (99/110, 90% versus (*vs*) 194/241, 81%; p<0.05) (Table 2).

**Table 2:** Clinical characteristics of NPEV associated with AFP case-patients, Uttar Pradesh, India, 2010–2011.

**Serotypes**	**E-1**	**E-2**	**E-3**	**E-4**	**E-6**	**E-7**	**E-9**	**E-11**	**E-12**	**E-13**	**E-14**	**E-15**	**E-18**	**E-19**	**E-20**	**E-21**	**E-24**
Number (n)	1	3	1	5	19	12	10	19	11	56	11	3	1	9	9	7	3
Mean Age (months)	14	58	31	44	40	40	40	43	41	48	28	26	48	53	40	29	45
Gender (Male/Female)	0/1	1/2	1/0	5/0	9/10	8/4	5/5	13/6	7/4	36/20	10/1	2/1	0/1	6/3	4/5	4/3	2/1
Fever at onset of paralysis No. (%)	-	3(100)	1(100)	3(60)	11(57)	5(41)	7(70)	12(63)	5(45)	31(55)	5(45)	1(33)	-	6(66)	7(77)	4(57)	-
Asymmetrical paralysis No. (%)	1(100)	3(100)	-	3(60)	15(78)	10(83)	7(70)	12(63)	7(63)	39(70)	11(100)	2(66)	1(100)	7(77)	9(100)	6(85)	3(100)
Residual Paralysis	-	-	-	2(40)	-	1(8)	1(10)	2 (10)	2(18)	8(14)	1(10)	-	-	-	1(11)	-	1(33)
**Serotypes**	**E-25**	**E-29**	**E-30**	**E-33**	**CV-B**	**CV-A14**	**CV-A20**	**EV-C95**	**CV-A24**	**EV-75**	**EV-101**	**EV-105**	**EV-107**	**CV-A9**
Number (n)	14	19	7	2	101	3	2	3	3	8	5	1	1	1
Mean Age (months)	38	44	37	27	53	61	51	13	90	53	32	42	24	38
Gender (Male/Female)	4/10	11/8	5/2	1/1	51/50	1/2	2/0	2/1	2/1	7/1	2/3	1/0	1/0	1/0
Fever at onset of paralysis No. (%)	10(71)	11(58)	5(63)	1(50)	57(56)	1(33)	2(100)	-	1(33)	5(63)	4(80)	1(100)	1(100)	1(100)
Asymmetrical paralysis No. (%)	11(78)	16(84)	7(88)	2(100)	90(89)	3(100)	2(100)	3(75)	3(100)	8(100)	2(40)	1(100)	1(100)	1(100)
Residual Paralysis	2(14)	2(11)	1(13)	-	5(5)	-	-	-	1(33)	2(25)	1(20)	-	-	-

E: Echovirus; EV: Numbered enterovirus, CV; Coxsackievirus

Numbers in brackets represent the percentage

#### Residual paralysis

Seventeen EV serotypes were found to exhibit paralysis status in a 60-day follow-up in 30 AFP patients, which included E-13 (n = 8), E-7, E-29, and CV-B5; EV-75, E-4, E-11, and E-12 (2 each) and E-9, E-14, E-24, E-30, CV-B3, CV-B4, CV-A24 and EV-101 (1 each) (Table 2). In this study, EV-101 and EV-75 were reported for the first time from AFP patients with residual paralysis.

#### Gender and age-dependent distribution of NPEV positive AFP cases

NPEV infections were detected in both males and females (213/1046, 20% vs 151/793, 19%; z = 0.62, p>0.05), with a male-to-female ratio of 1.4:1. These were found to be comparable in children aged less than 2 years and the age group greater than two to less than 6 years. Of the total 364 NPEV-positive AFP patients, the children aged >6 to <15 years showed significantly lower NPEV positivity than children aged ≥2 to <6 years (75/545, 13.8% vs 183/838, 21.8%; z = 3.76, p< 0.0001).

#### Seasonal distribution of NPEV-associated AFP cases

NPEV-positive AFP cases were detected throughout the year, and a significantly greater number of cases were found during June-September (monsoon months) (194/700, 28%) than in March-May (summer months) (115/521, 22.1%; z = 2.18, p<0.05), October–November (post monsoon months) (41/287, 14%; z = 4.58, p<0.05) and December–February (winter months) (14/331, 4%; z = 8.99, p<0.00001).

#### Geographical distribution of NPEV- associated AFP cases

The geographic distribution of the NPEVs reflected their circulation in 27 districts of Uttar Pradesh, with a high percentage of NPEV-positive AFP cases seen in the Sitapur region, followed by the Kheri, Hardoi, Lucknow, Unnao, Kannauj and Raebareli regions located in the central part of the state.

#### Impact of vaccination on positivity of NPEV-associated AFP cases

The effect of the number of OPV doses in the AFP cases positive for NPEVs in the <2 to <15 age group was also investigated. It was found that the majority of the NPEV-positive AFP (218/364, 60%) patients who were 5 years of age accounted for the largest proportion of patients who received high doses of OPV in the range of >7–39+ doses (Table 3). However, the least number of NPEV patients were in the >6 to <15 years group who received more than 7 doses of OPV. The analysis of the results by chi-square test showed high significance (χ^2^ = 281, p = 0.001).

**Table 3:** Effect of OPV doses on positivity of NPEV infected AFP cases.

No. of OPV doses	No. (%) of NPEV positive AFP cases among different age groups						
	>2yr	≤2->4yr	≤4->6 yr	≤6->8 yr	≤8->10 yr	≤10->12 yr	≤12->15 yr
0–9	57(55)	5(4)	2(4)	2(5)	-	3(22)	2(22.2)
10–19	46(44)	74(60)	3(5)	2(5)	2(14)	3(22)	3(33.3)
20–29	1(1)	44(36)	32(54)	12(32)	7(50)	3(22)	2(22.2)
30–39^+^	-	-	22(37)	18(47)	1(7)	3(22)	2(22.2)

Abbreviation: OPV, oral polio vaccine; yr: Year;

The number inside the bracket represents the percentage of NPEV positive AFP cases in each category

### Comparison of the nucleic acid of the study sequences

Phylogenetic analysis of the E-13 partial VP1 sequences shows the existence of two clear genetic lineages appearing on two different clades; the largest branch contains related strains in clades A, B, and C, while the second divergent E-13 clade is labeled D. Both E-13 lineages contain antecedent E13 strains mainly from Central and South Asia (Bangladesh, Pakistan, and earlier India strains). The representative isolates of the most common E-13 genotypes, were subclustered during phylogenetic analysis (Fig 1) and were initially identified as untypable isolates in the MNT test. However, when filtered and further subjected to typing using twofold concentrated intersecting antiserum pools (RIVM, Netherlands) to compare the results from the old antigenic serotyping method with results from serotyping (genotyping) using partial VP1 sequences, 6 of 37 isolates were neutralized and confirmed as E-13, while 31 isolates remained unneutralised.

**Fig 2:**
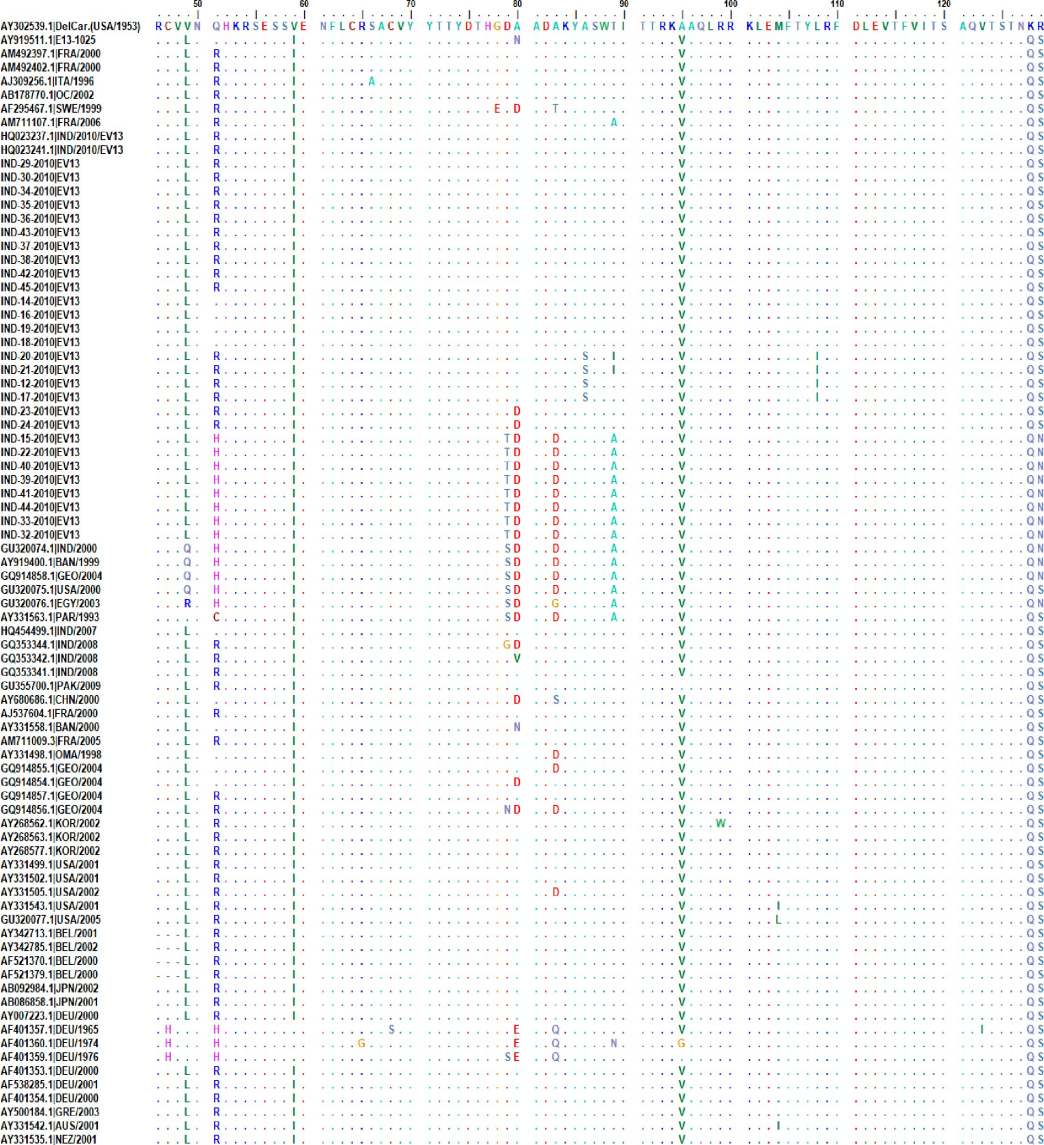
Alignment showing comparison of deduced amino acid sequences of 84 residues covering the BC loop of the highly variable VP1 region of EV13 isolates. The BC loop (encompass 12 amino acids, 78–89 AA) is boxed and “.” in alignment denote amino acid residue conservation with the Echovirus 13/Del Carmen prototype strain (AY302539.1).

The deduced amino acid sequences of the VP1 region of all E-13 study isolates of different clades, the prototype strain (E-13/Del Carmen) and other E-13 reference strains were aligned and compared. For both typeable and untypable isolates among Clade-A and Clade-C, no amino acid variation in the antigenic neutralization site 1, the BC loop region was identified but existed outside of the BC loop (Fig 2).

The untypable isolates among Clade-B and Clade-C showed amino acid substitutions in the BC loop of the VP1 protein, with four substitutions at positions 79, 80, 83 and 89 within the BC loop. Threonine (T) at amino acid position 79 appeared to be fixed in the studied isolates, which was not detected in other E-13 strains (Fig 2). This result indicated that the neutralization epitopes for untypable isolates identified among these subtypes might exist on sites other than the BC loops of the VP1 protein, as a number of important neutralization epitopes other than the BC-loop may also exist on VP1, VP2 and VP3 proteins [18, 19].

**Fig 3:**
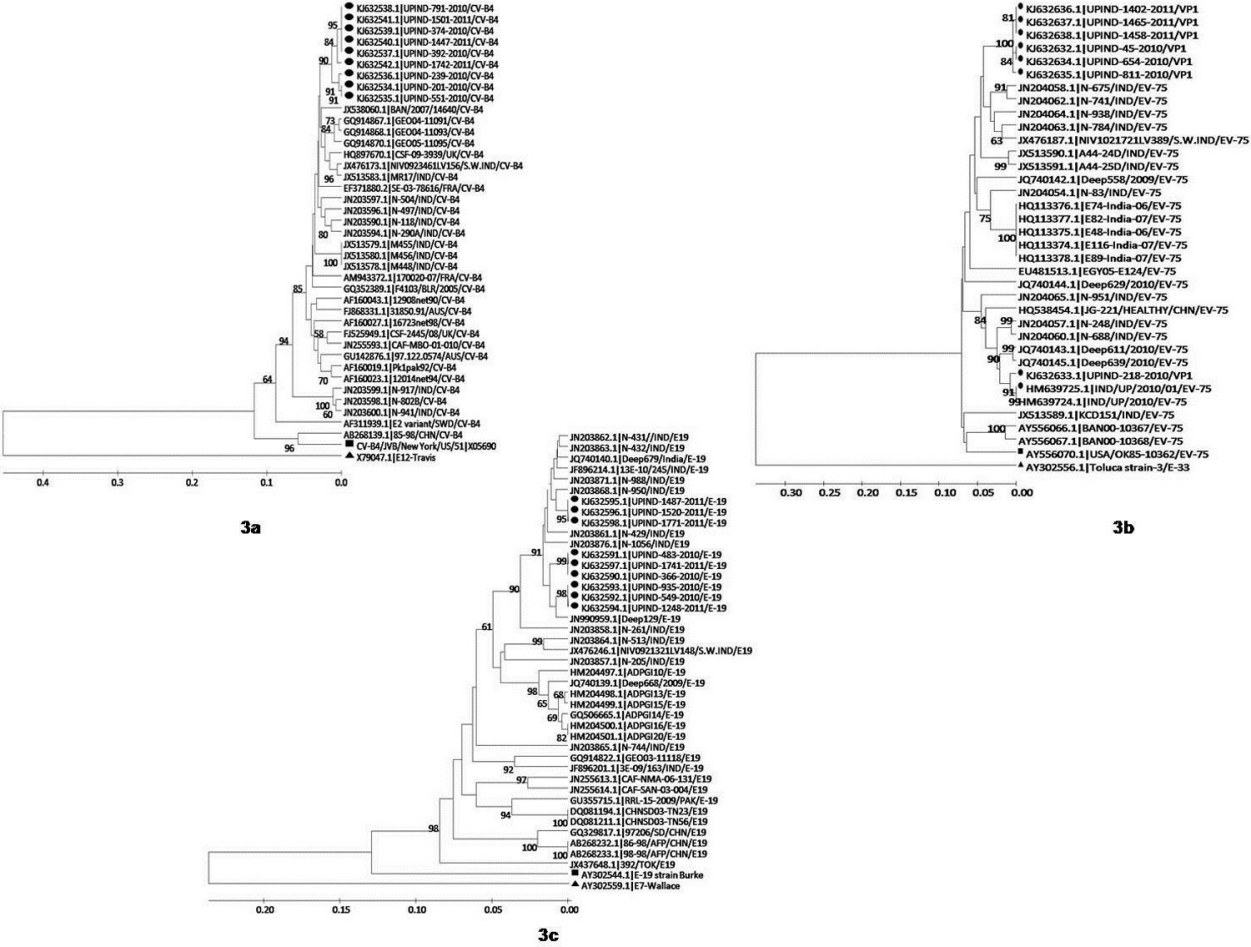
**Phylogenetic tree based on the partial VP1 region of isolates of serotypes of (a) CV-B4, (b) E-19, (c) EV-75 from this study**, indicated by black-closed circle, (●) and other reference strains are available from the Genbank database. Triangle (▲) indicates out group and black closed square box (■) as prototype strains The trees were constructed by UPGMA (unweighted pair group method using arithmetic averages) using maximum composite likelihood nucleotide substitution model with statistical significance of the phylogenetic analyses estimated by bootstrap analysis with 1,000 pseudoreplicate datasets. Bootstrap values >60% are indicated on the trees. Scale bar indicates nucleotide substitutions per site.

Although phylogenetic analysis of the enterovirus strains from UP revealed a high degree of genetic diversity within a serotype, the clusters and subcluster appear to be closely related to the recently identified strains from India. However, for the UP CV-B4 clusters, they were genetically more distant and grouped together with strains from Georgia and Bangladesh, suggesting the recent importation of this cluster into the region (Fig 3A). The CV-B isolates of the study, although grouped according to serotypes CV-B1 to CV-B6, represented a different cluster and subcluster within each type (S1 Fig). Of the 8 EV-B75 study isolates, six were monophyletic and closely related to one another, exhibited 99% to 100% nucleotide identity (NT ID), and reflected a common origin. These isolates were clustered closely together with strains recently identified from AFP patients, diarrhoea patients and encephalitis patients in southern and northern India. The two EV-B75 isolates exhibited 11% to 13% nucleotide divergence from the other six study strains in tree topology, occurred on a separate branch and were grouped separately together with contemporary strains from India and China (Fig 3B). Thus, the finding suggests that more than two different EV-B75 genotypes are in circulation in the region.

Phylogenetic analysis of the VP1 sequences of E-19 strains revealed that the UP E-19 strains, (n = 9) with 96% to 100% NT IDs among them, were grouped together in a separate cluster (bootstrap value of 91%) and displayed NT IDs in the range of 96% to 98% with few of the other recently E-19 strains isolated from encephalitis patients, environmental specimens and AFP patients from different parts of India. However, these UP E-19 isolates also exhibited more than 10% nucleotide divergence from other Indian strains, suggesting the existence and circulation of more than one genotype of E-19 in India. The phylogenetic analysis revealed that the study strains with different genotypes together with other Indian strains form a separate large cluster, which differs from those detected in other countries (Fig 3C). It is noteworthy here that the study strains in analysis displayed 85.2–88.4% NT IDs with the recently established new genotype of E-19 detected in Pakistan [20].

For the EV-B101 isolates (n = 5), the closest matches were from strains isolated in the Central African Republic (CAF) and Bangladesh (2007). To the best of our knowledge, the EV-B101 study strains have been identified for the first time from India from AFP, which previously included only two sequences in the NCBI database from a West African country [21]. The VP1 nucleotide sequences of a single strain, UPIND-1511-2011, in this study were the second EV-B107 strain reported from India (87% NT ID), which shared 86% NT ID with the EV-B107 prototype strain TN94-0349 (AB426609) from Thailand.

**Fig 4:**
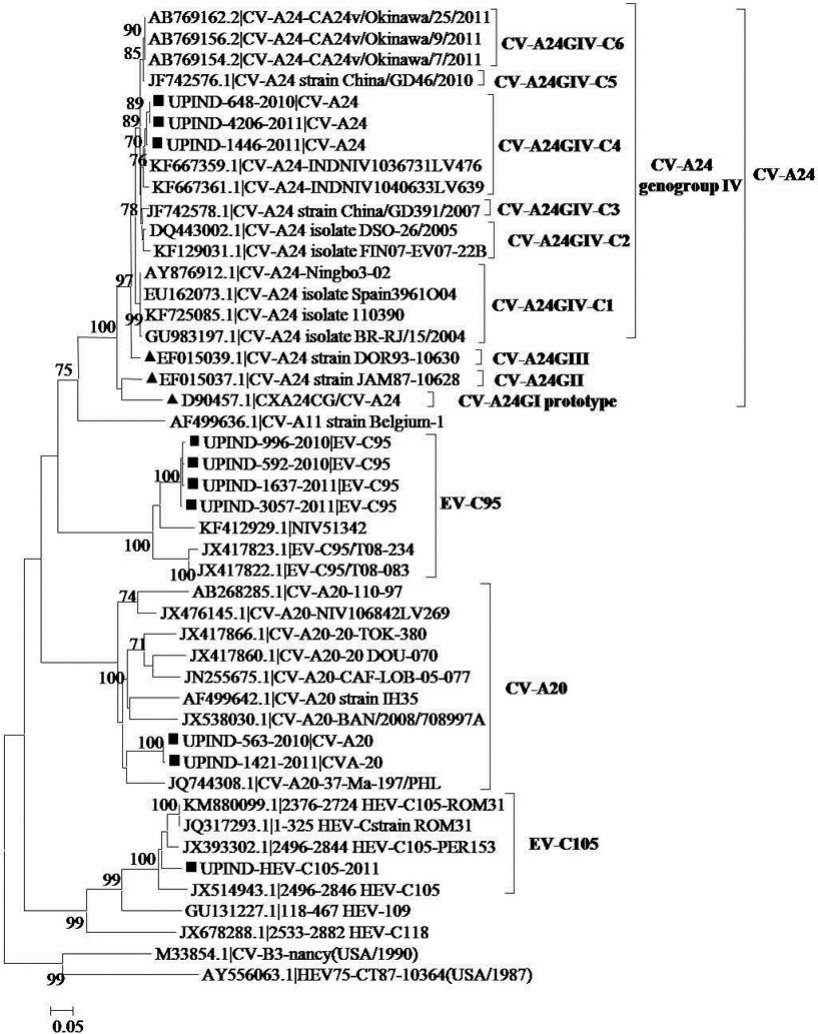
Phylogenetic analysis of partial VP1 region of nucleotide sequences of EV serotypes of EV-C species from this study. (●), Black-closed circles represent EV-C serotypes from this study, while, black closed square boxes (■), represent all EV-C prototypes. The neighbor-joining tree was generated by using MEGA 5 software with statistical significance of the phylogenetic analyses estimated by bootstrap analysis with 1,000 pseudoreplicate datasets. Scale bar indicates number of nucleotide substitutions per site.

In VP1 sequencing analysis, the EV-C isolates were assigned to four different serotypes as CV-A20, CV-A24, EV-C95 and EV-C105 (Fig 4). Phylogenetic analysis revealed the clustering of four field isolates of EV-C95, UPIND-996-2010, UPIND-1637-2011, UPIND-592-2010 and UPIND-3057-2011, together with the two strains, T08-083 and T08-234, recently typed as EV-C95 with a bootstrap value of 100% and displayed 83% to 85% pairwise percent NT IDs (98% to 99% amino acid identity) with the available EV-C95 strains from Chad, a sub-Saharan African country. The four EV-C95 strains isolated for the first time in India in this study represent the rare newly identified enterovirus serotypes, which have so far been reported only from sub-Saharan Africa [22]. The CV-A24 study strains (n = 3) clustered together with the genogroup GIV strains shared the highest sequence identities with the other Indian strains from distant geographical regions in a genetic cluster, C4, and include strains that had been found circulating among AFP patients in the southwestern part of India (Fig 4). According to the VP1 sequence analysis, one of the EV-C study strains belonged to the recently detected novel EV-C105 serotype. The study isolate, UPIND-EV-C105-2011, was in a monophyletic clade cluster close together with the recently described genotypes such as EV-C105, EV-C109, EV-C117 and EV-C118 on a separate EV-C subbranch, supported with a higher bootstrap value of 99% and reported for the first time in India (Fig 4).

The data in the study suggest that most of the UP EVs evolved to form a distinct lineage within a given serotype that differs from those prevalent and recently identified in India (and other countries).

## Discussion

During the post-polio eradication era, the rate of isolation of NPEVs remains a clinical yardstick for the surveillance of acute flaccid paralysis (AFP) cases in the field. Polio surveillance will remain a high priority for the foreseeable future, but, in addition, the detection and identification procedures for NPEV associated with AFP need to be prioritized. The burden and severity of AFP associated with NPEV in India are largely unknown but are suspected to be high, as polio accounts for only a small percentage (<1%) of the total suspected viral AFP cases [4]. The work presented in this study provides the first comprehensive investigation of clinical, epidemiological and molecular features of NPEVs associated with nonpolio AFP in the Uttar Pradesh State, northern India.

In the study, the total NPEV positivity detected through cell culture isolation in the years 2010 and 2011 was 75% (183/244) and 77% (92/120), respectively, higher in children 5 years of age and compared to the high positivity reported by other studies among NP-AFP children [6]. The percentage of NPEV isolates typed by the MNT method was comparable to the percentage of EVs seroneutralized in other studies [10, 23]. Among the serotyped isolates, CV-B and Echovirus (E)-11, E-12, E-13 and E-6 were the most frequent NPEV serotypes isolated from AFP patients and healthy children in the present study. Similar studies from India and elsewhere are available, which have reported on the frequent isolation of these serotypes but with a tendency of re-emergence at variable intervals for a limited period [10, 23]. However, 55% of the NPEV isolates in this study remained untypable during seroneutralization, which is consistent with the previous studies [24, 25]. Failure of neutralization resulted in UT-EV isolates, likely due to the absence of homologous antibodies in the pools used, the aggregation of virions, the presence of a virus mixture and the presence of the new serotype [26].

The UT-EV isolates generated in the study were genetically characterized by molecular serotyping. Encouraged by the initial rapid typing results emanated and previously published by us [27], 188 UT-EV isolates in this study were molecular serotyped by direct VP1 genotyping from stool specimens. The three UT-EVs that exhibited mixed infection by >1 strain involving two different serotypes were resolved through cloning and sequencing. However, this process is labour-intensive and unsuitable for the screening of a large number of samples. VP1 RT-PCR and sequencing of EV mixtures can also be problematic, *e*.*g*., unreadable sequences, or the identification of different EV types from the individual forward and reverse primer sequencing reactions [28]. Nevertheless, VP1 RT-PCR and sequencing is a better methodological choice for rapid NPEV recovery from stool samples. This could be a feasible approach and realistic for monitoring the prevalence of NPEV from AFP samples. For those isolates (n = 4) in the study that did not grow in cell culture, next-generation sequencing technologies could serve as the most promising and reliable new methods for the identification and complete genome characterization of novel or uncultivable enteroviruses [29].

The present study has revealed the predominance of EV-B species followed by EV-C and EV-A species, consistent with the previous findings [12, 21]. Identification of EV-B as the prominent EVs in the study indicates that this genetic group seeks close attention, as an upsurge in the rate of EV-B species-related cases of neurological manifestation has been recently observed growing in the region [30]. No EV-D strain was isolated in the present study. It has been shown that the use of HEp-2c cells increases the number of EV-C isolates, not EV-D. EV-Ds are rarely isolated in this type of study [21].

None of the cell lines other than RD was used for NPEV isolation in the present study; however, applying a combination of two or three different cell lines with two passes in cell culture makes the method slow, rigorous and resource consuming. The lower isolation rate of EV-C species in the present study might be attributed to the absence of the Hep-2 cell line in the isolation technique. The use of Hep-2 cell lines in the isolation techniques in the EV surveillance could be realistically important to monitor the burden of AFP borne by NPEVs, as shown in other studies [21, 22]. Of particular interest were the EV-C serotypes such as CV-A20, CV-A24, EV-C95 and EV-C105 in this study and detection of EV-C in healthy children from the same geographical region indicates the circulation of EV-C species in this polio-endemic region [31, 32]. Previous studies have shown that the cocirculation of both PV and non-PV EV-C strains provided favourable conditions for the generation of recombinant vaccine derived polioviruses (VDPVs) with increased virulence [22, 33]. Since 2009, at least 50 cases of VDPV have been detected in India; fortunately, none of them were types of any circulating VDPVs (cVDPVs) [34]. Thus, to avert any kind of fulminant outbreak situation in the post-polio eradication era due to recombinant cVDPV, molecular investigation of enteroviruses should be undertaken in the existing AFP surveillance system that would address the burden associated with nonpolio AFP.

In the present study, EV-C105 serotypes have been identified for the first time in India from the fecal specimens of patients with AFP that produced negative results in cell culture but positive by rRT-PCR [35]. Though a recent study has shown EV-C105 association with AFP [36], further studies are needed to ascertain their pathogenic role in causing AFP.

EV isolates identified in the present study were assigned into 34 different serotypes, including recently reported new serotypes such as EV-B75, EV-B101, EV-B107, EV-C95 and EV-C105. During recent years, numbered enterovirus serotypes have been identified from different parts of India [10, 12, 13, 30]. The detection of these serotypes from distant geographical regions suggests their widespread distribution in India, with low levels of demonstrated activity; however, further studies are required to understand their role in the aetiology of neurological disorders. Previous studies from India and Pakistan have identified 6–15 serotypes in AFP cases [6, 10], but recent studies from India have observed an increase in detection in EV serotypes [12, 13]. The high frequency of EV serotypes might be attributed to high infection pressure in these highly populated areas of India and adoption of molecular methods for virus detection.

Of the 34 EV serotypes in the study, E-13 has been the predominant serotype identified, while in another report from southern India by Rao et al. [13], E-13 was the second most predominant serotype detected during the years 2007–2009 in AFP cases. Thus, for five years (2007–2011), the E-13 serotype has shown a continuous pattern of circulation in India, likely among nonimmune persons. The genetically diverse E-13 strains were arranged into different clusters in the study when seroneutralized with concentrated antisera, and most of the E-13 isolates, including some isolates with identical partial VP1 amino-acid sequences, failed to get neutralized.

Virus neutralization (VN) tests in cell culture can fail for many reasons [26, 37]. A common problem encountered is that multiple EV serotypes grow in a single cell culture. One solution to this problem is plaque purification of the “isolate” in cell culture, which itself is tedious and can lead to many more VN tests than originally planned. In addition to E-13, other serotypes, such as CV-B1, CV-B3, CV-B5, CV-B6, E-2, E-6, E-7, E-11, E-14, E-19, E-20, E-25, E-33 and EV-75, identified in this study with variation in the number of isolates in each year have been frequently reported during 2007–2011 in the state of Uttar Pradesh, suggesting the existence of these viruses as the most common in the region. However, changes in the constitution of the predominant serotype have occurred over this period, indicating that a different temporal pattern was exhibited by the individual serotypes, which might have been accompanied by outbreaks of enteroviral illness [38]. Therefore, the concomitant circulation and temporal distribution of enteroviruses in cases of neurological illnesses, healthy children and in the environment underscore the importance of the establishment of a continuous EV surveillance (both clinical and environmental) system in the region for a better understanding of the transmission dynamics and epidemiology of the enterovirus infections.

In contrast to previous studies, low rates of isolation of enteroviruses in healthy children have been reported in this study, which could be explained due to the small number of contact AFP patients represented in the healthy children cohort that fail to share exposure, and consequently viral flora, with AFP cases [22]. In the present study, no significant difference was inferred for the isolation of NPEV in healthy children, which could be explained by the high availability of an immunologically susceptible cohort in young children. The EV serotypes, such as E-6, E-11, E-12, E-13, E-19, E-25, CV-B3 and CV-B4, belonging to EV-B species were uncovered for both AFP patients and healthy children. Moreover, the EV-B80 isolate was identified only in healthy children. Few of the serotypes isolated from healthy individuals with EV infection may lead to AFP [39].

High population density, poor sanitation and multiple infections with entero-pathogens removes the immunogenicity for oral poliovirus vaccine (OPV) against poliomyelitis and influences the capacity of NPEVs to remain in silent circulation in healthy and susceptible populations. Different serotypes within the same enterovirus species have been detected in AFP children in the present study and it is well known that different serotypes or genotypes within the same enterovirus species have been associated with different symptoms [39].

The higher predominance of EV infections in male children was observed. This is comparable to the detection rates reported elsewhere [6, 12] and might be ascribed to males encountering more opportunities to participate in public activities that put them at a higher risk of infection. The analysis of the available clinical data of the study revealed that 57% of NPEV-infected AFP patients exhibited fever at the onset of paralysis. This fever symptom showed no significant association with NPEV infection in the study. Nonetheless, the clinical findings for nine AFP-cases with the isolation of E-4, E-9, E-11, E-13, E-20, E-25, EV-75 and CV-B5 had a fever at the time of the onset of paralysis accompanied by asymmetrical paralysis and residual paralysis, which mimicked polio-like illness. Although not definitive, the NPEV isolation from stool samples of AFP suggests the involvement of this agent in the aetiology. Numerous reports have provided evidence for the role of NPEVs in AFP [5, 10, 24]. NPEVs have been detected in epidemics of paralytic disorder [8].

The age distribution of enterovirus-associated illness in a surveillance report from the United States (1970–2005) has shown that most of the cases were reported in young children [40]. The present study shows that the majority of the NPEV-associated AFP cases occurred in children less than 5 years of age. Isolation of NPEV decreased significantly with an increase in age, reflecting a high level of exposure or susceptibility at a young age. The high NPEV-positive NP-AFP cases from the central part of the state of UP with high population density suggests the likely association of NP-AFP rate with the population density. Immunologically susceptible cohorts and nonhygienic environmental conditions probably facilitated the spread and maintenance of these viruses in high density human populations [38].

Variation in the isolation rate of NPEV was observed throughout the study period. Higher NPEV positivity in AFP cases was observed during the monsoon months and significantly lower NPEV positivity was noted in the cool climate during winter months. A high degree of humidity together with elevated temperatures may have favoured the high EV transmission during the monsoon months [10]. In recent years, epidemiological studies from Uttar Pradesh have shown that enterovirus infection identified in different clinical categories follows the pattern of transmission similar to other studies from India, much more likely to elucidate the actual patterns of enteroviruses circulating in the Indian subcontinent [12, 13, 30, 31].

The incidence of AFP in the present study was found to be directly proportional to the number of OPV doses received, as shown in Table 3. This finding is concurrent with the AFP cases, where those who had received greater than seven doses of OPV accounted for the largest proportion of NPEV positive AFP cases, and perhaps the increase of OPV doses may induce the strain shifts of entero-pathogens. It is well evident that over the years, when the high incidence of WPV was recorded under PV surveillance, the high percentage of EVs and the emergence of new EVs have been indicated [10, 11, 14]. Gradually, the intensive efforts at polio eradication have likely resulted in the shift to pathogens from classical polio to NPEVs [41].

The phylogenetic data in the present study suggest that few of the prevalent serotypes evolve to form a distinct lineage within a given serotype that differ from those prevalent and recently identified serotypes in India and other countries [13, 27]. The study strain sequences depicted in Fig 3 show similarity with strains present in different geographical regions of India. Many of the sequences in the study share high similarity with the other sequence data available in the northern region from studies related to enterovirus diseases such as meningitis and encephalitis (Fig 3) and thus together represent a part of the cluster and subcluster in circulation, which is different from other regions of the country [12, 13, 30, 31].

In conclusion, this study reveals the circulation and the identification of a high degree of genetically diversified NPEVs belonging to lineages isolated in distant countries and of different clusters in co-circulation. The data presented in the study also underline the importance of the analysis of epidemiological and genetic characteristics of NPEV infections in gaining better insights into the disease burden of nonpolio AFP caused by NPEV. The epidemiologic and genetic information gathered warrants further studies in larger groups of both AFP and healthy children from both polio endemic and polio-free geographical regions of India to identify the etiologic NPEV type(s) associated with sporadic or epidemic AFP in the post-polio eradication era. Thus, incorporating molecular EV surveillance in the high-risk areas for polio in the post-polio elimination phase would be of clinical significance in the long-term assessment of clinical outcomes and providing a better understanding of the severity and the spectrum of the NPEV associated with NP-AFP cases.

## Supporting information

S1 Fig Phylogenetic analysis of partial VP1 region of coxsackievirus B1-B6 nucleotide sequences. CV-B1-CV-B6 serotype from this study is represented by black-closed circles, (●). Where, black closed square boxes (■), represent all CV-B prototypes. The neighbor-joining tree was generated by using MEGA 5 software with statistical significance of the phylogenetic analyses estimated by bootstrap analysis with 1,000 pseudoreplicate datasets. The prototype strain of coxsackievirus A (CV-A) 16 was used as an out-group. Scale bar indicates number of nucleotide substitutions per site.
(TIF)
