# Supplementary figures and images for: Identification and characterization of nonpolio enterovirus associated with nonpolio-acute flaccid paralysis in polio endemic state of Uttar Pradesh, Northern India
Source: PLoS One. 2019 Jan 30;14(1):e0208902. doi: 10.1371/journal.pone.0208902 (PMC6353074; doi:10.1371/journal.pone.0208902)

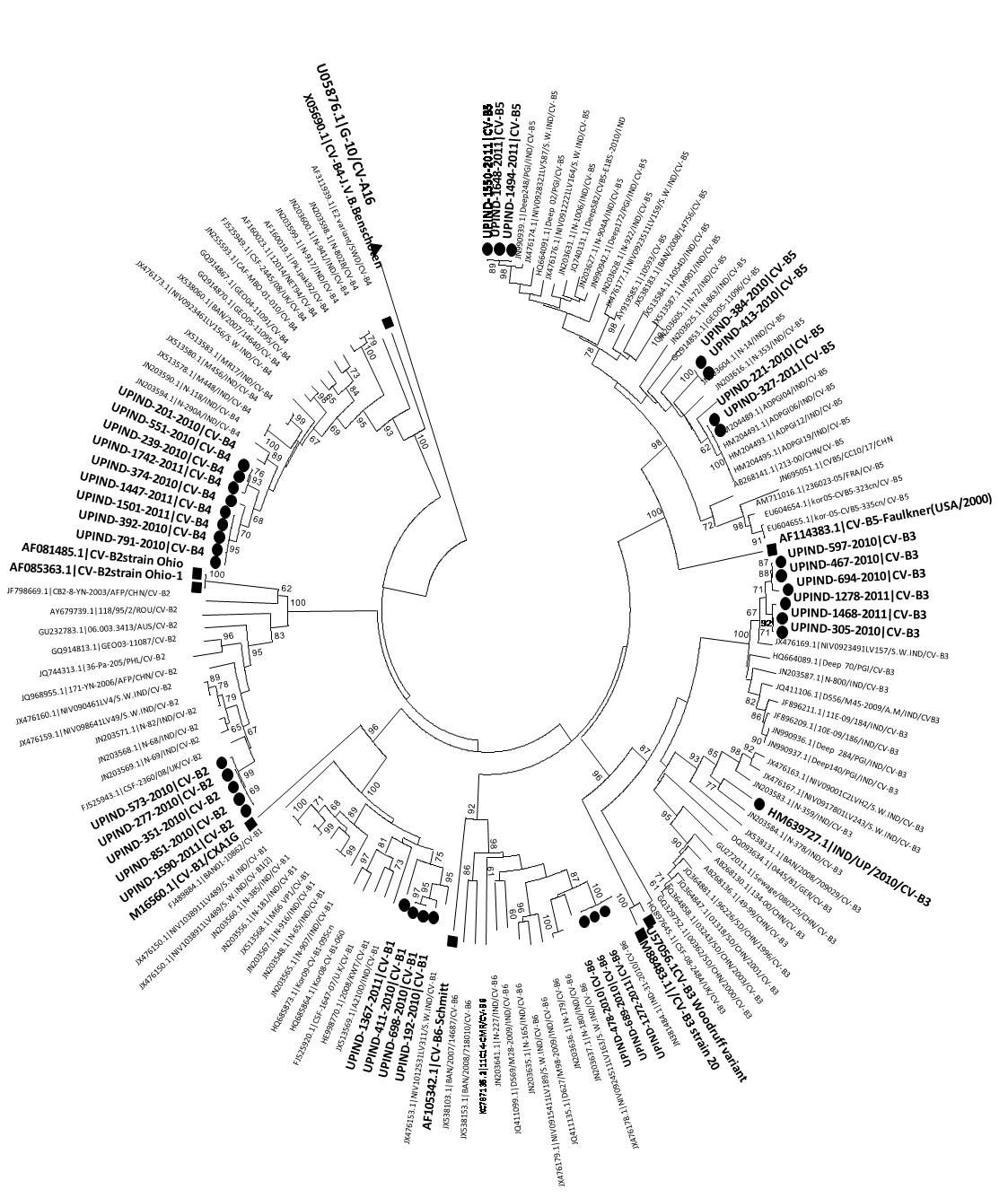

Supplement: S1 Fig — CV-B1-CV-B6 serotype from this study is represented by black-closed circles, (●). Where, black closed square boxes (■), represent all CV-B prototypes. The neighbor-joining tree was generated by using MEGA 5 software with statistical significance of the phylogenetic analyses estimated by bootstrap analysis with 1,000 pseudoreplicate datasets. The prototype strain of coxsackievirus A (CV-A) 16 was used as an out-group. Scale bar indicates number of nucleotide substitutions per site. (TIF) [file pone.0208902.s001.tif]
